# Rabbit Hemorrhagic Disease Virus Non-structural Protein 6 Induces Apoptosis in Rabbit Kidney Cells

**DOI:** 10.3389/fmicb.2018.03308

**Published:** 2019-01-09

**Authors:** Mengmeng Chen, Xing Liu, Bo Hu, Zhiyu Fan, Yanhua Song, Houjun Wei, Rulong Qiu, Weizhong Xu, Weifeng Zhu, Fang Wang

**Affiliations:** Key Laboratory of Veterinary Biological Engineering and Technology, Ministry of Agriculture, National Center for Engineering Research of Veterinary Bio-products, Institute of Veterinary Medicine, Jiangsu Academy of Agricultural Sciences, Nanjing, China

**Keywords:** rabbit hemorrhagic disease virus, non-structural protein 6, 3C-like protease, apoptosis, rabbit hemorrhagic disease

## Abstract

Rabbit hemorrhagic disease (RHD) is a highly contagious disease caused by rabbit hemorrhagic disease virus (RHDV). Previous research has shown that RHDV induces apoptosis in numerous cell types, although the molecular mechanisms underlying the apoptosis induced by RHDV are not well understood. One possible factor is non-structural protein 6 (NSP6), a 3C-like protease that plays an important role in processing viral polyprotein precursors into mature non-structural proteins. To fully establish a role for NSP6, the present study examined the effects of ectopic expression of the protein in rabbit (RK13) and human (HeLa and HepG2) cells. We found that NSP6 suppressed cell viability and promoted apoptosis in all three cell types in a dose-dependent manner. We also identified increased caspase-3, -8, and -9 activities in RK13 cell, and an increased Bax to Bcl2 mRNA ratio. Mechanistically, the ability of NSP6 to induce apoptosis was impaired by mutation of the catalytic His27 residue. Our study has shown that RHDV NSP6 can induce apoptosis in host cells and is likely an important contributor to RHDV-induced apoptosis and pathogenesis.

## Introduction

Rabbit hemorrhagic disease (RHD) is a highly contagious disease characterized by acute liver damage and disseminated intravascular coagulation (DIC) (Alonso et al., 1998; Jung et al., 2000). The first outbreak of RHD was reported in 1984 in the Jiangsu Province of China (Zhu et al., 2016) and has quickly spread to most parts of the world (Calvete et al., 2018; Dalton et al., 2018). This has led to the deaths of millions of rabbits and hares, representing a serious threat to their populations. The etiologic agent that causes RHD is rabbit hemorrhagic disease virus (RHDV) (Abrantes et al., 2012), a calicivirus of the *Lagovirus* genus. RHDV contains both genomic RNA (gRNA) and additional subgenomic RNA sequences (sgRNA). The genomic RNA consists of a positive-sense single-stranded RNA molecule that is 7437 nucleotides in length and includes two slightly overlapping open reading frames (ORFs), ORF1 and ORF2. ORF1 encodes a large polyprotein that is cleaved into mature non-structural proteins 1–6 (NSP1–6) and the major structural protein (VP60) (Meyers et al., 1991, 2000). Of the non-structural proteins, NSP6 is known to be a trypsin-like cysteine protease (Allaire et al., 1994; Boniotti et al., 1994; Wirblich et al., 1995) that is released from larger precursors molecules via proteolytic cleavage at the N and C termini. Mutagenesis analysis has suggested that three amino acids in particular (His27, Asp44, and Cys104) play an important role in NSP6 function as a catalytic triad (Wirblich et al., 1995).

Although the first outbreak of RHD was more than 30 years ago, the mechanisms underlying its pathogenesis are still not fully understood. The liver is believed to be the main site of RHDV reproduction, with viral replication leading to liver cell apoptosis and necrosis (Jung et al., 2000). It is also known that systemic hemorrhagic diathesis and DIC can lead to rabbit death. These are most likely consequences of liver cell loss through RHDV-induced apoptosis (Alonso et al., 1998; Trzeciak-Ryczek et al., 2015). Hepatocytes are indeed the first choice cells in this study, but so far there is no stable rabbit liver cell line, which brings some difficulties to the research. Studies have shown that RHDV infection can not only cause liver cell apoptosis, but also cause the apoptosis of multiple cells in various organs such as heart, spleen, lung, kidney, etc., (Alonso et al., 1998). Therefore, a stable kidney cell line from rabbit (RK13 cell) was selected as the object to study cell apoptosis in this research. Studies have also shown that macrophages and endothelial cells also display the morphological hallmarks of apoptosis (Gelmetti et al., 1998). Further research has found that granulocyte and lymphocyte apoptosis also occurs in rabbits infected with various RHDV strains (Marques et al., 2010; Niedzwiedzka-Rystwej and Deptula, 2012; Teixeira et al., 2012; Niedzwiedzka-Rystwej et al., 2013). More recently, studies have indicated that N-acetyl cysteine (San-Miguel et al., 2006), cardiotrophin (Tunon et al., 2011b), and melatonin (Tunon et al., 2011a), can attenuate liver damage and prolong survival in RHDV-infected rabbits. This is likely due to the induction of various anti-apoptotic factors and their inherent anti-apoptotic effects. Altogether, these findings suggest that apoptosis plays an important role in RHD-mediated symptoms and may be a key determinant of disease pathogenesis. However, the exact viral components that contribute to the apoptosis-inducing effects of RHDV are unknown, and research is hampered by the fact that RHDV cannot be propagated in cell culture.

To determine which viral components may be involved in RHD pathogenesis, our study employed ectopic expression of NSP6 in rabbit and human cells to identify any effects on apoptosis and cell viability. We first amplified NSP6 from the full-length cDNA clone of RHDV strain NJ-2009 through PCR and cloned the product into a pcDNA3.1-6 His vector for expression and subsequent analysis. This showed that NSP6 expression could induce apoptosis in RK13, HeLa, and HepG2 cells, likely via modification of caspase expression and altered Bax and Bcl2 expression ratios. This effect was ameliorated by mutation of the catalytic His27 residue of NSP6, suggesting involvement of this residue in NSP6 apoptosis induction. Altogether, our findings indicate that NSP6 contributes to RHDV-induced apoptosis of host cells and is therefore central to RHDV pathogenesis in rabbits.

## Materials and Methods

### Cell Culture

*Oryctolagus cuniculus* (rabbit) kidney cells (RK13), human liver cancer cells (HepG2) and human cervical cancer cells (HeLa) were purchased from the American Tissue Culture Collection (ATCC; Manassas, VA, United States). They were cultured in Dulbecco’s modified Eagle’s medium (DMEM; Gibco; Thermo Fisher Scientific, Waltham, MA, United States) supplemented with 10% fetal bovine serum (FBS; Gibco) in a humidified atmosphere of 95% air and 5% CO_2_.

### Plasmid Construction

Genes encoding wild-type NSP6 were amplified using PCR from the full-length cDNA clone of RHDV strain NJ-2009. Single-point mutant plasmids (targeting the catalytic triad of His27, Asp44, and Cys104) were constructed using a Mut Express II Fast Mutagenesis Kit V2 (Vazyme Biotech, Nanjing, China). The primer pairs used are shown in Table 1. The genes were then cloned into the multiple cloning site of a pcDNA 3.1-6 His vector (GenScript, Nanjing, China). The resultant plasmids were designated pcDNA 3.1-NSP6, H27N, D44G, and C104G. The plasmids were used to transfect the RK13 cells with Lipofectamine 3000 reagent (Invitrogen; Thermo Fisher Scientific), according to manufacturer’s instructions. Untransfected cells and vector pcDNA 3.1-6 His transfected RK13 cells were used as mock and vector controls, respectively.

**Table 1:** Sequences of primers used for amplification of NSP6 and the generation of NSP6 point mutants.

Name	Sequence
NSP6-Fwd	5′-GCGGAATTCCACCATGGGCCTACCTGGTTTTATGAG-3′
NSP6-Rev	5′-GCGAAGCTTCATTACACCATCACAAAGGG-3′
H27N -Fwd	5′-CTCCAACACC**C**ATACTGCCAGGTCAAGTTGCTCAGAGATT-3′
H27N -Rev	5′-CTGGCAGTAT**T**GGTGTTGGAGATGTACAGTCCGTTCCCTA-3′
D44G -Fwd	5′-CCCACCACTG**G**CCTGTGCCTTGTCAAGGGCGAGACAATCC-3′
D44G -Rev	5′-AAGGCACAGG**C**CAGTGGTGGGTGAGCATGTGACAATCTCT-3′
C104G -Fwd	5′-CCACGGGGAC**G**GTGGACTACCGTTGTATGACTCTAGTGGG-3′
C104G -Rev	5′-GGTAGTCCAC**C**GTCCCCGTGGGTGGTCTGCGTGCAACCAT-3′


### Indirect Immunofluorescence Assay (IFA)

RK13 cells were transfected with pcDNA3.1-NSP6, H27N, D44G, C104G, or pcDNA 3.1-6 His vector for 12 h in 6-well culture plates and then rinsed with phosphate-buffered saline (PBS). The cells were then fixed with chilled 95% ethanol for 10 min at 4 C and washed three times with PBS plus Tween-20 (PBST). Cells were incubated with an anti-His monoclonal antibody (1:1000 in PBST; GenScript) for 1 h at 37°C, washed, and then a secondary dye-coupled antibody (FITC-conjugated goat anti-mouse IgG; Southern Biotech, Birmingham, AL, United States) was added at a dilution of 1:10000 and incubated at 37°C for 1 h. After rinsing five times with PBS, the plates were analyzed via fluorescence microscopy (Zeiss, Oberkochen, Germany).

### Cell Viability Assay

An MTT cell proliferation and cytotoxicity assay kit (Beyotime, Shanghai, China) was used to detect any potential effects of NSP6 on cell proliferation according to manufacturer’s instructions. Assays were performed 24 and 48 h post transfection (hpt) using the previously described lipofectamine protocol. Following the final incubation step with formazan, optical density was determined using a microplate reader (Bio-Rad Laboratories, Hercules, CA, United States). The relative cell number of transfected RK13 cells was compared with that of pcDNA3.1-6 His vector or pcDNA3.1-NSP6. Each experiment was performed in triplicate.

Trypan blue dye exclusion assay: RK13 cells were transfected with pcDNA3.1-NSP6 and cell viability was determined by 0.4% trypan blue solution staining using automated cell counter (JIMBIO, China) at 24 h, 48 h and 72 hpt. Empty vector-transfected RK13 cells are shown as controls.

### Terminal Deoxynucleotidyl Transferase dUTP Nick End Labeling (TUNEL) Assay

A TUNEL assay kit (Roche, Basel, Switzerland) was used to detect DNA fragmentation in response to apoptotic stimuli according to the manufacturer’s instructions. The cells were then analyzed by fluorescence microscopy for TUNEL staining (Zeiss).

### Apoptosis Analysis

For apoptosis analysis, 5 × 10^5^ cells (including RK13, Hela, and HepG2 cells) were seeded onto plates and transfected with recombinant plasmids (including pcDNA3.1-NSP6 and mutants) or empty vector for 24 h. The cells were then washed with PBS and resuspended in 100 μL binding buffer supplemented with 5 μL annexin V (10 μg/mL) and 5 μL propidium iodide (PI) (20 μg/mg) for 10 min in the dark. Next, 400 μL binding buffer was added, and the samples were immediately screened using flow cytometry to assess annexin V staining and permeability.

### Caspase-3, -8, and -9 Activity

Caspase-3 -8, and -9 activities were measured using colorimetric assay kits (Beyotime) according to manufacturer’s instructions. RK13 cells transfected with pcDNA3.1-NSP6 and pcDNA3.1-6 His vector for 24 h were harvested by centrifugation and incubated in lysis buffer on ice for 5 min. The lysate was then centrifuged at 15,000 rpm and 4 C for 15 min and the final protein content was determined using a Pierce BCA protein assay kit (Thermo Fisher Scientific). Next, 20 μg of protein was incubated for 2 h at 37 C with DEVD-pNA colorimetric substrate, IETD-pNA, and LEHD-pNA for the caspase-3, 8, and 9 assays, respectively. Activity was estimated by measuring absorption at 405 nm and subtracting the background values obtained from wells without colorimetric substrate.

### Quantification of Bcl2 and Bax mRNA Levels

RK13 cells were transfected with NSP6 for 12, 24, or 36 h. Total RNA was then extracted using the Total RNA Kit I (Omega Bio-Tek, Norcross, GA). Isolated RNA (1 μg) was then used for subsequent cDNA synthesis using a PrimeScript RT Reagent Kit (TaKaRa Bio, Kusatsu, Japan). Specific primers for rabbit Bcl2 and Bax were used for real-time PCR analyses with SYBR Master Mix (TaKaRa Bio) and an ABI7300 Real-Time PCR System (Applied Biosystems, Foster City, CA, United States). Bcl2 and Bax mRNA levels were normalized to glyceraldehyde-3-phosphate dehydrogenase (GAPDH) expression, and data are presented as fold change relative to the medium-only control. Primers used for real-time PCR are listed in Table 2.

**Table 2:** Primers used for real-time PCR.

Name	Sequence	GenBank accession no.
Bcl2-F	5′-GATTGTGGCCTTCTTTGAGTTC-3′	XM_008261439.2
Bcl2-R	5′-AAGTCTTCAGAGACACCCAGGA-3′	
Bax-F	5′-TTTGCTTCAGGGTTTCATCC-3′	XM_002723696.3
Bax-R	5′-GGCAGCGATCATCCTCTGTA-3′	
GAPDH-F	5′-GAATCCACTGGCGTCTTCAC-3′	L23961.1
GAPDH-R	5′-CGTTGCTGACAATCTTGAGAGA-3′	


### Statistical Analysis

All experiments were performed independently in triplicate. Significant differences between groups were determined with one- or two-way analysis of variance (ANOVA) using GraphPad Prism 5.0 (GraphPad Software, Inc., San Diego, CA, United States). A threshold *P*-value of 0.05 was considered statistically significant.

## Results

### The Expression of NSP6 in RK13 Cells

**FIGURE 1:**
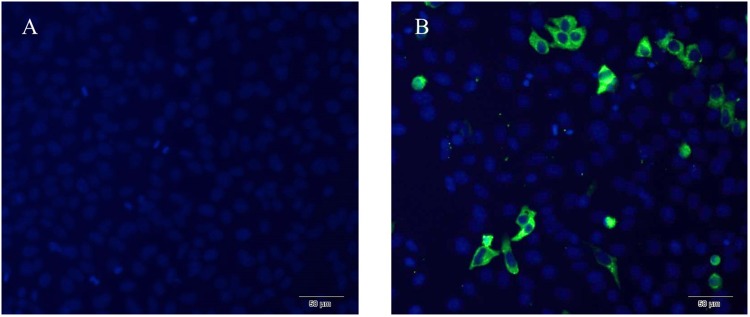
Recombinant RHDV-NSP6 proteins were expressed in RK13 cells. **(A)** Representative image of untransfected controls showing no immunofluorescence. **(B)** RHDV His-NSP6 fusion proteins in transfected RK13 cells at 12 hpt was determined by indirect immunofluorescence assay using FITC-conjugated murine anti-His monoclonal antibodies (green). The nuclei were counterstained with DAPI (blue).

The expression of NSP6 in transfected rabbit kidney cells (RK13) was determined using a His-NSP6 fusion protein and immunofluorescent staining (Figure 1). The fluorescent signal was observed in the cytoplasm of pcDNA3.1-NSP6-transfected cells at 12 hpt and was found primarily aggregated in the cytoplasm. No fluorescent signaling was detected in the control.

### NSP6 Affects RK13 Cell Growth

**FIGURE 2:**
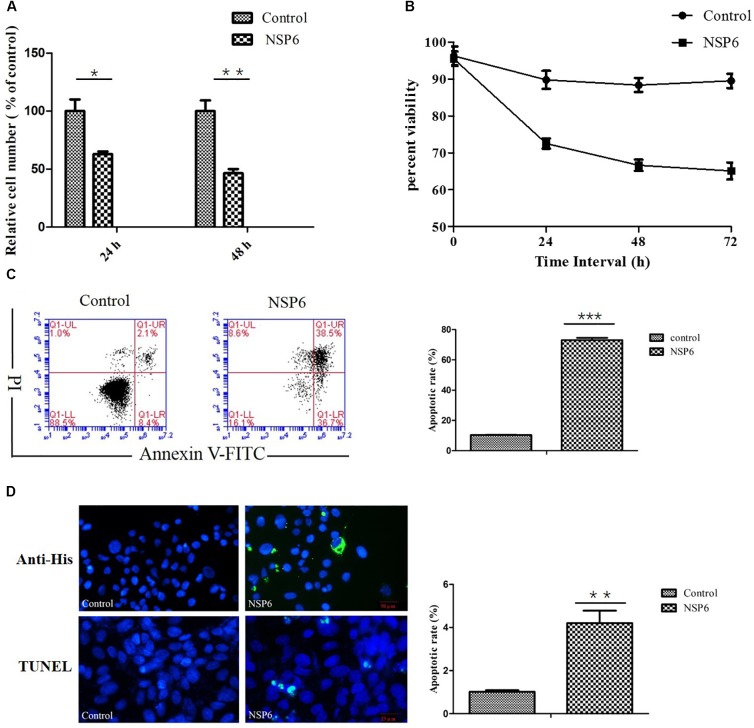
RHDV-NSP6 influenced cell growth and apoptosis of RK13 cells. **(A,B)** Cell viability analysis of RK13 cells. RK13 cells transfected with pcDNA3.1-NSP6 (5000 ng) was detected by MTT assay **(A)** and trypan blue dye exclusion assay **(B)** at the indicated time points post transfection. Empty vector transfected RK13 cells were used as control. **(C)** Cell apoptosis was measured by flow cytometry analysis. Results are expressed as scatter diagram (left) and calculated percentage of annexin-V-positive cell population (right). **(D)** TUNEL labeling of NSP6-transfected RK13 cells. Empty vector and NSP6-transfected RK13 cells fixed at 24 hpt were labeled with TUNEL (green) and then counterstained with DAPI (blue). NSP6 expression was also determined by immunofluorescence using FITC-conjugated murine anti-His monoclonal antibodies (green). Scale, 50 μm. Results are from one representative of three independent experiments. ^∗^*P* < 0.05; ^∗∗^*P* < 0.01; ^∗∗∗^*P* < 0.001.

To assess the effect of NSP6 on RK13 cell growth, RK13 cells were assayed using MTT and trypan blue dye. In MTT assay, RHDV-NSP6 reduced the relative cell number of RK13 cells to 63 and 46% of control cell at 24 and 48 hpt (Figure 2A), respectively. In trypan blue dye exclusion test, the viable cell rate of RK13 cells transfected with NSP6 reduced to 72, 66 and 65% at 24, 48 and 72 hpt (Figure 2B), so there is a slight discrepancy between the results of MTT assay and trypan blue dye exclusion test. The reason for the difference in results may be the different experimental principles. Trypan blue dye is used to examine cell viability by detecting cell membrane integrity, while in MTT assay the activity of mitochondrial reductase enzymes is used as an indicator of cell viability. Above all, both of the results indicate NSP6 expression may affect cell metabolism reducing growth of the cells.

### NSP6 Induces Apoptosis in RK13, HeLa, and HepG2 Cell Lines

**FIGURE 3:**
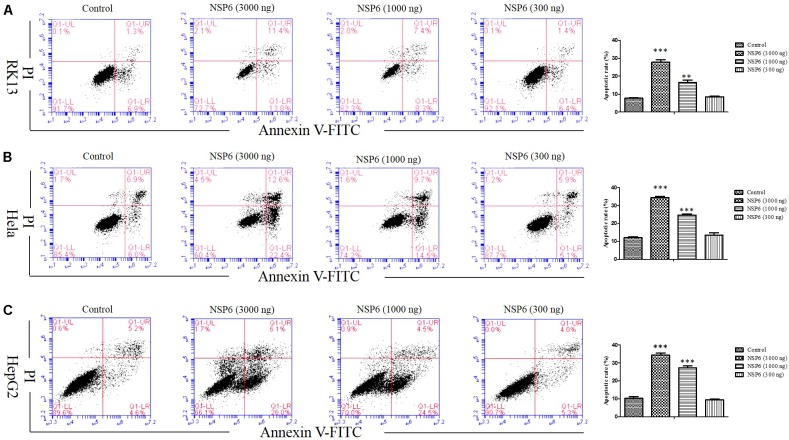
RHDV-NSP6 induced apoptosis in dose-dependent manner. **(A)** RK13, **(B)** Hela, and **(C)** HepG2 cells were transfected with NSP6 recombinant plasmid or vector (3000 ng). 24 h after transfection, the apoptosis rate were detected by annexin V/PI double staining combined with flow cytometry. The doses of pcDNA3.1-NSP6 are 3000, 1000, and 300 ng, respectively. Results are from one representative of three independent experiments. ^∗∗^*P* < 0.01; ^∗∗∗^*P* < 0.001.

To examine if the decrease in cell viability induced by NSP6 was due to increased apoptosis in host cells, we transfected RK13 cells with either empty pcDNA3.1-6 His or pcDNA3.1-NSP6 plasmids and analyzed apoptosis via annexin V/PI staining with flow cytometry. As showed in Figure 2C, 24 h after transfection with pcDNA3.1-NSP6, the percentage of apoptotic cells was markedly increased to 75% (including 30% early apoptotic rate and 45% late apoptotic rate). This result presented a difference comparing dates presented in Figures 2A,B. The main reason for this difference is that the results from both MTT and trypan blue dye exclusion test reflect the level of living cells, but cells under way in apoptosis may still counted as living cells in those method. However, in annexin V/PI assay, early apoptotic cells and late apoptotic cells were detected. In agreement with these observations, a TUNEL analysis further confirmed the presence of an apoptotic cell population in pcDNA3.1-NSP6 transfected RK13 cells. As shown in Figure 2D, compared to vector control, TUNEL-positive cells (green) were observed in pcDNA3.1-NSP6-transfected RK13 cells. However, only adherent cells were detected in TUNEL assay as a large number of apoptotic cells in the supernatant were discarded with the culture medium during the experiment. Therefore, the apoptotic rate indicated by TUNEL assays was only 4%. In addition, we found that percentage of apoptotic RK13 cells induced by NSP6 was increased in a dose-dependent manner (Figure 3A). Moreover, similar results were also obtained in human HeLa and HepG2 cells (Figures 3B,C), indicating that the effect was not limited to RK13 cells and that NSP6-induced apoptosis is not cell specific.

### NSP6 Promotes Activation of Caspase-3, -8, and -9

**FIGURE 4:**
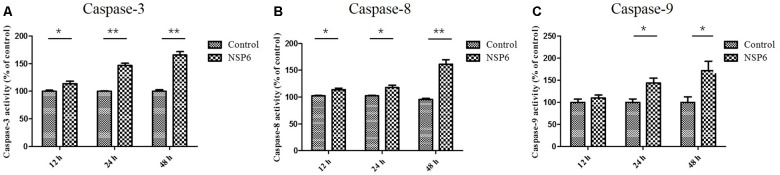
RHDV-NSP6 enhances the activity of caspase-3, 8, and 9 in RK13 cells. RK13 cells were transfected with pcDNA3.1-NSP6 or empty vector (3000 ng). Activity kinetics of **(A)** caspase-3, **(B)** caspase-8, and **(C)** caspase-9 was measured at the indicated time points post transfection using a caspase-3/8/9 assay kit. Results are from one representative of three independent experiments. ^∗^*p* < 0.05; ^∗∗^*p* < 0.01.

Caspases are cysteine proteases that play fundamental roles in the apoptotic responses of cells to different stimuli. To gain insight into the mechanism of NSP6-induced apoptosis, we examined the activities of caspase-3, caspase-8, and caspase-9. These proteins are key components of the caspase cascade and are central to the intrinsic and extrinsic pathways of apoptosis. We found that caspase-3 activity was first observed in NSP6-transfected cells at 12 hpt and upregulated 1.6-fold at 48 hpt (Figure 4A). Furthermore, the activities of caspase-8 and caspase-9, which are representative initiator to caspases in the death receptor-mediated and mitochondrial apoptotic pathways, respectively, were also measured. As shown in Figures 4B,C the caspase-8 and caspase-9 activities were also increased in NSP6-transfected cells and maximal increases were observed at 48 hpt (1.6-fold and 1.7-fold increases in activity, respectively, when compared with that of the pcDNA3.1-6 His vector control). This is consistent with the increase in apoptotic cells observed in pcDNA3.1-NSP6-transfected cells using annexin V/PI flow cytometry and TUNEL.

### Relative Expression of Bcl2 and Bax mRNA in NSP6 Transfected RK13 Cells

**FIGURE 5:**
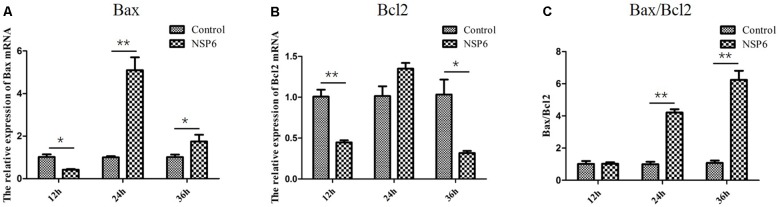
The level of Bax and Bcl2 mRNA and Bax/Bcl2 ratio largely changed in RK13 cells. RK13 cells were transfected with pcDNA3.1-NSP6 or empty vector (3000 ng). Cells were harvested and processed at the indicated time points post transfection. The mRNA expression of **(A)** Bax and **(B)** Bcl2 mRNAs were detected by quantitative real-time PCR. **(C)**The Bax/Bcl2 ratio was also analyzed. Results are from one representative of three independent experiments. ^∗^*p* < 0.05; ^∗∗^*p* < 0.01.

Previous research has shown that N-acetyl cysteine can attenuate liver damage and prolong survival in RHDV-infected rabbits (San-Miguel et al., 2006). This protective effect is related to the apoptosis regulator Bcl-2, a family of evolutionarily related proteins. These proteins govern mitochondrial outer membrane permeabilization and can be either pro-apoptotic (Bax) or anti-apoptotic (Bcl2). We studied the effects of NSP6 on these proteins and found Bax expression was also significantly higher in NSP6 transfected cells at 24 and 36 hpt when compared with the vector control (Figure 5A). The expression of Bcl2 was significantly lower in NSP6-transfected cells at 12 and 36 hpt (Figure 5B) relative to vector control. Overall, there was an increased ratio of Bax to Bcl2 in NSP6 transfected cells compared with that of vector control (Figure 5C), indicating that NSP6-induced cell apoptosis involves Bcl-2 family members.

### His27 Is Important for NSP6-Induced Apoptosis

**FIGURE 6:**
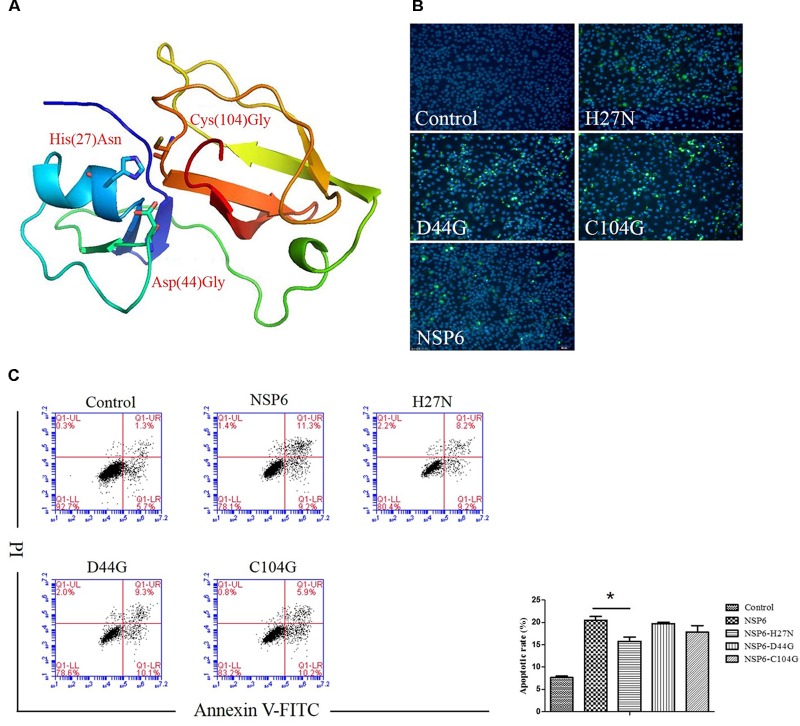
The effect of NSP6 mutants in RK13 cells. **(A)** Schematic representation of the positions of the mutant residues His27Asn (H27N), Asp44Gly (D44G), and Cys104Gly (C104G) in NSP6. **(B)** Mutants and wild-type NSP6 expression (green) in RK13 cells was determined by immunofluorescence at 12 hpt. Nuclei were counterstained with DAPI (blue). Images were taken at × 200 magnification under a ZEISS fluorescence microscope. **(C)** Analysis of apoptosis in RK13 cells induced by NSP6 and mutants. RK13 cells transfected with pcDNA3.1-NSP6, H27N, D44G, C104G, or control vector (3000 ng) stained with Annexin V/PI were analyzed by flow cytometry at 24 hpt. Results are from one representative of three independent experiments. ^∗^*P* < 0.05.

NSP6 is a 3C-like serine protease that contains a canonical catalytic triad of His27, Asp44, and Cys104. To assess if these residues are involved in NSP6-triggered apoptosis, three single-point mutants, His27Asn (H27N), Asp44Gly (D44G), and Cys104Gly (C104G), were generated in the wild-type NSP6 protein. A diagram of the protein mutant residue positions is illustrated in Figure 6A. Initially, protein expression for each mutant was confirmed using indirect immunofluorescence (Figure 6B). Next, the apoptosis rates induced by each mutant were analyzed by flow cytometry using annexin V/PI staining of RK13 cells. As shown in Figure 6C, all mutants induced some degree of apoptosis. However, the level of apoptosis induced by the H27N mutant was significantly lower than that of wild-type NSP6, implying that His27 took part in the apoptosis induction.

## Discussion

Apoptosis plays an important role in the pathogenicity of a wide variety of viruses (Garg et al., 2012; van den Berg et al., 2013; Okamoto et al., 2017; Elizalde et al., 2018; Guo et al., 2018). Like many other positive-strand RNA viruses, RHDV-infection can also induce cell apoptosis *in vivo* and is believed to contribute to RHD pathogenesis (Alonso et al., 1998). Previously, RHDV research was limited due to the lack of a stable cell system to culture the virus *in vitro*. So far, it has remained unclear which viral components contribute to RHDV-induced apoptosis. RHDV NSP6 is a 3C-like protease. Functionally, RHDV NSP6 is similar to the 3C proteases of picornaviruses (Wirblich et al., 1995), although much more is known of the picornavirus genome. These proteases show serine protease activity and are structurally homologous to cellular serine proteases of the trypsin family. The cleavage reactions they mediate are required for releasing the different functional viral polyproteins. In addition to this protease activity, it has been reported that picornavirus 3C proteases, including those from coxsackievirus B3, enterovirus 71, and poliovirus, can also induce apoptosis (Barco et al., 2000; Li et al., 2002; Calandria et al., 2004; Lin et al., 2006; Zaragoza et al., 2006; Chau et al., 2007). The role of 3C-like protease induced apoptosis has also been characterized in severe acute respiratory syndrome-associated coronavirus (Lin et al., 2006). However, whether RHDV NSP6 with similar structure can induce apoptosis has not been reported.

In this study, RHDV-NSP6 was cloned into pcDNA3.1-His vector. His-NSP6 fusion protein was found primarily aggregated in the cytoplasm of RK13 cells detected by immunofluorescence assay (Figure 1). This is slightly different with the results from Urakova et al. (2015). Urakova team perform research about subcellular localization of recombinant non-structural RHDV proteins. Their study pointed out the NSP6 (RHDV protease) was found to accumulate in nuclear and cytoplasmic compartments at 24 h after transfection (Urakova et al., 2015). We speculated that one reason for this discrepancy may be due to the different time points we choosed. In our experiment, we found that very few NSP6+ positive cells was detected and a large number of cells occured apoptosis and shed into the culture medium without detection at 24 hpt by indirect immunofluorescence assay. Therefore, we changed the detection time and detected the expression of NSP6 protein in RK13 cells at 12 hpt. Another reason is the limitations of our method. We did not analyze the fluorescence signal in the nucleus at 12 hpt. Trypan blue test showed that a decrease in cell viability observed in pcDNA3.1-NSP6 transfected RK13 cells which was further confirmed by MTT assay. Both of the tests showed that NSP6 protein is cytotoxic to the cells, and the cytotoxicity was time dependent (Figures 2A,B). To confirm the decrease in cell viability was due to the induction of apoptosis, the quantification of cellular apoptosis was performed by flow cytometry and TUNEL assay. We found a marked increase in TUNEL positive cells in pcDNA3.1-NSP6 transfected RK13 cells (Figure 2D) and the increased number of annexin V+ cells (lower right quadrant, Figure 2C) and AnnexinV+/PI+ cells (upper right quadrant, Figure 2C) confirmed induction of apoptosis by pcDNA3.1-NSP6 in RK13 cells. In addition, NSP6 induced apoptosis in a dose-dependent manner in RK13 cells (Figure 3A). This was replicated in human HeLa and HepG2 cells (Figures 3B,C), indicating that the effect is not limited to RK13 cells. Based on the above results, we speculated and NSP6 is highly likely to be responsible for the apoptosis observed during RHDV infection.

Caspase-3 is a major effector caspase in both the extrinsic and intrinsic apoptotic pathways acting on enzymes that are indispensable for chromatin condensation and DNA fragmentation (Duprez et al., 2009; Lan et al., 2013). A previous studies has shown a marked increase in caspase-3 activity at 36 h (12.5-fold) and 48 h post-inoculation (12.6-fold) in RHDV-infected animals, indicating apoptosis activation (Garcia-Lastra et al., 2010). Consistent with these results, our current study found that caspase-3 activity were activated in NSP6-transfected cells, indicating that NSP6 induces RK13 cells apoptosis through activation of the effector caspase, caspase-3. Furthermore, we found that the caspase-8 (representative initiator caspase in the death receptor-mediated apoptotic pathway) and caspase-9 (representative initiator caspase in mitochondrial apoptotic pathway) activities were also increased in NSP6-transfected cells. These results suggest that RHDV NSP6 induced caspase-dependent apoptosis in RK13 cells via both the death receptor-mediated and mitochondrial apoptotic pathways. However, the activation of the initiator caspase-8 can also be related to the mitochondrial apoptotic pathway via cleavage of Bid (a member of the pro-apoptotic Bcl-2 family) and translocation of the truncated Bid to mitochondria (Wang et al., 1996; Lan et al., 2013). Therefore, to clarify the detailed mechanisms of NSP6-induced apoptosis, further investigations that focus on the kinetics of signal transduction factors are necessary. Conversely, Bcl2, an anti-apoptotic protein that promotes cell survival (Polcic et al., 2017), acts to regulate the intrinsic and extrinsic pathways through broad molecular interactions with other critical cellular proteins. Meanwhile, Bax, a protein that also features a Bcl-2 like conserved domain, plays a vital role in promoting programmed cell death. The balance in Bax and Bcl2 expression can affect mitochondrial cytochrome c release, promoting apoptosis during increased Bax to Bcl2 ratios (Pepper et al., 1997; Li et al., 2017). A study also suggested that apoptosis induced by RHDV-infection is related to the modulation of Bcl2 and Bax genes (San-Miguel et al., 2006). This is in agreement with our results where the relative expression levels of Bcl2 and Bax mRNA changed significantly in NSP6 expressing cells. More importantly, we found that the ratios of Bax to Bcl2 were higher in NSP6 expressing cells, further promoting apoptosis.

The amino acids His27, Asp44, and Cys104 play an important role in NSP6 function as a catalytic triad (Wirblich et al., 1995). Asn and Gly were chosen as residue substitutions, as they do not have similar structural functions to the wild-type residues, cannot form disulfide bridges, and are different in size and chemistry and could thus damage proteolytic activity (Boniotti et al., 1994). So three single-point mutants were constructed and the apoptosis induction of each mutant were confirmed by flow cytometry. Our data showed that mutation of the 3C-like protease active site (specifically the His27 residue) impaired NSP6-induced apoptosis indicate that the mechanism of RHDV NSP6 promoted apoptosis is similar to the 3C proteases of picornaviruses. Moreover, our result is consistent with previous research showing that mutating sites involved in protease activity impairs apoptosis induced by an enterovirus 71 3C protease in human neural cells (Li et al., 2002).

In conclusion, our findings demonstrate that RHDV NSP6 significantly contributes to cell apoptosis and suggest that the protein alone is sufficient to induce apoptosis in RK13, HeLa, and HepG2 cells. This appears to be linked to the activation of caspase-3, -8 and -9, and increase in Bax to Bcl2 ratios, which promotes apoptosis. In addition, we have shown that a His27 residue in the catalytic domain of the protein is involved in the apoptotic effect. Thus, NSP6 is important in the apoptosis induced by RHDV and is a central mediator of RHD pathogenesis.

## Author Contributions

MC designed and performed the experiments and analyzed the data. MC and BH wrote the manuscript. XL, ZF, YS, HW, WZ, RQ, and WX assisted in performing some experiments. FW contributed essential ideas and discussion. All authors read and approved the final manuscript.

## Conflict of Interest Statement

The authors declare that the research was conducted in the absence of any commercial or financial relationships that could be construed as a potential conflict of interest.
